# Creating Music With Fuzzy Logic

**DOI:** 10.3389/frai.2020.00059

**Published:** 2020-10-30

**Authors:** Rodrigo F. Cádiz

**Affiliations:** ^1^Faculty of Arts, Music Institute, Pontificia Universidad Católica de Chile, Santiago, Chile; ^2^Department of Electrical Engineering, School of Engineering, Pontificia Universidad Católica de Chile, Santiago, Chile

**Keywords:** fuzzy logic, computer music, machine learning, sound synthesis, parametric control, algorithmic composition

## Abstract

Fuzzy logic is an artificial intelligence technique that has applications in many areas, due to its importance in handling uncertain inputs. Despite the great recent success of other branches of AI, such as deep neural networks, fuzzy logic is still a very powerful machine learning technique, based on expert reasoning, that can be of help in many areas of musical creativity, such as composing music, synthesizing sounds, gestural mappings in electronic instruments, parametric control of sound synthesis, audiovisual content generation or sonification. We propose that fuzzy logic is a very suitable framework for thinking and operating not only with sound and acoustic signals but also with symbolic representations of music. In this article, we discuss the application of fuzzy logic ideas to music, introduce the Fuzzy Logic Control Toolkit, a set of tools to use fuzzy logic inside the MaxMSP real-time sound synthesis environment, and show how some fuzzy logic concepts can be used and incorporated into fields, such as algorithmic composition, sound synthesis and parametric control of computer music. Finally, we discuss the composition of *Incerta*, an acousmatic multichannel composition as a concrete example of the application of fuzzy concepts to musical creation.

## 1. Introduction

Music, although considered a science by many, is not an exact science, but rather a collection of qualities, ranging from the emotional to the intellectual in varying degrees (Suiter, 2010a). Several concepts in music are not absolute but rather relative and its terminology is not entirely precise. Many musical concepts do not possess an absolute meaning, and composers, with a few notable exceptions, do not specify every detail about how their musical creations should be converted into sound. For example, a slow tempo indication in a musical score can be interpreted very differently by different analysts or performers. Indeed, many musical attributes are described by imprecise (or fuzzy) concepts, such as *presto, forte, piano, andante*, or *allegro*. León and Liern (2012) provide the music of J.S. Bach as an example of such a fuzzy approach to composing, as in his music features, such as the instrumentation or the tempo are not explicitly stated in the scores.

Following the same line of argument, authors, such as Milicevic (1999) state that music, unlike language, is fuzzy, while others, such as León and Liern (2012) consider a musical score to be a truly fuzzy system, meaning that performers are required to execute very complex actions based on uncertain concepts written in the score. If we accept this premise, some aspects of fuzzy logic theory seem to be a natural way of predicting the aesthetic outcomes of music (Suiter, 2010a) and its structure.

Fuzzy logic (Kosko, 1993; McNeill and Freiberger, 1993; Cox, 1994; Bandemer and Gottwald, 1995; Klir and Yuan, 1995; Yen and Langari, 1999) is a branch of artificial intelligence specifically designed to handle imprecise and vague concepts. Fuzzy logic can be conceived as a logical system based on a more general concept of truth, one that is not two-valued (true or false) and very appropriate for reasoning under uncertainty, by allowing different degrees of membership or several values of truth. In general, the application of fuzzy logic inference to a problem emulates some aspects of human reasoning, for example, the quantification of imprecise information or making decisions given unclear or partial data (Kosko, 1993).

Artificial intelligence aims to construct computational algorithms that can perform some level of reasoning and exhibit problem-solving skills similar to those of humans. Fuzzy logic has an additional objective: “to explore an effective trade-off between precision and the cost in developing an approximate model of a complex system or function” (Yen, 1999). However, perhaps one of the most important qualities of fuzzy logic concerning music-making is its capacity for modeling non-linear systems without the need of explicitly constructing a complex mathematical model. Indeed, as Suiter (2010a) states: “A significant feature of music is that the aesthetic outcome is often more than the sum of its technical elements. Indeed, what is the role of timber, attack, duration, decay, articulation, spatialization, register, texture, voicing, entries and timing, rhythm, tempo, or meter? What does musical form, structure, or process contribute? In fact, it is often the means and details of the interactions between the distinct elements which significantly influence the effectiveness of the whole work. This means music is, technically, a non-linear system.”

This article is structured as follows. First, we briefly introduce the main ideas and concepts behind fuzzy logic and its application, with an emphasis on the fuzzy approximation theorem and fuzzy inputs as latent spaces. Second, we conduct an updated survey of the utilization of fuzzy logic in musical applications. Third, we present the Fuzzy Logic Control Toolkit (FLCTK), a set of tools to generate musical content in the MaxMSP real-time sound synthesis environment. Fourth, we provide detailed examples of applications in sound synthesis, algorithmic composition, and many-to-many musical mappings. Fifth, we discuss some compositional aspects of *Incerta*, an acousmatic multichannel composition done in MaxMSP with the FLCTK. Finally, some conclusions and future lines of work are presented.

## 2. Fuzzy Logic

Zadeh (1965) introduced the concept of fuzzy sets, which are different from standard sets in the sense that they operate with *multi-valued logic*. Compared to other, perhaps more popular, artificial intelligence techniques, fuzzy logic is simpler and more flexible, making it a very appealing tool for musical applications. Indeed, fuzzy logic systems have found applications in a great multiplicity of fields, notably engineering and control applications (Kosko, 1993; Klir and Yuan, 1995), but also in areas apparently unconnected, such as data analysis (Bandemer and Gottwald, 1995), economics, business, and finance (Von Altrock, 1997), sociology (Dimitrov and Hodge, 2002), or geology (Demicco and Klir, 2004). Fuzzy logic algorithms can be easily found in everyday popular objects, such as cameras, camcorders, or washing machines, but also on unmanned vehicles, such as trains.

### 2.1. The Fuzzy Principle

Kosko (1993) coined the phrase *everything is a matter of degree* to emphasize a key element of fuzzy logic theory. In fuzzy logic, inputs and outputs are fuzzified, meaning that their values belong in varying degrees to several fuzzy sets. For example, if we consider the sound intensity range of 30–120 dB, and we want to determine whether a given intensity is low, medium, or high, does a value of 90 dB correspond to a high intensity? As it is closer to 120 than to 30 perhaps, but there are other values which are higher in intensity. Therefore, instead of assigning only one label to it, it is not a bad idea to consider a fuzzified version of this concept, one in which this particular value belongs in different degrees to both the medium and high intensity labels. In consequence, fuzzy sets are not exclusive, they allow *partial membership* of its elements. Unlike traditional crisp logic, where elements belong or do not belong to a particular set, in fuzzy logic an element of the set can be a member of it only partially. In this way, fuzzy logic handles uncertain terms and partial values of truth. Elements are not entirely black or white; they can acquire any shade of gray. Mathematically, this implies membership values between 1 and 0.

### 2.2. Fuzzy Sets

As we previously stated, a fuzzy set contains members to some degree (Kosko, 1993). Let *F* be a fuzzy set with an universe of discourse *X* = {*x*}, defined as the mapping μ_*F*_(*x*) : *X* → [0, α]. The universe of discourse is the range of all possible real scalar values of some measurement or items of information that we want to fuzzify. This mapping assigns to each *x* a value in the range [0, α]. When α = 1 the set is called *normal*. A fuzzy set contains a distribution, also called *membership function*. When a distribution is of zero width, the membership function collapses to a singularity, which corresponds to the traditional case of a crisp set. If these singularities can only have one of two possibilities, they perform binary logic. μ_*F*_ is called the grade of membership or *degree of truth* of *x*. Fuzzy sets, although usually modeled after triangular or Gaussian distributions, can adopt any form, and no shape has been proven to be the best (Mitaim and Kosko, 2001).

#### 2.2.1. Fuzzification and Defuzzification

**Figure 1:**
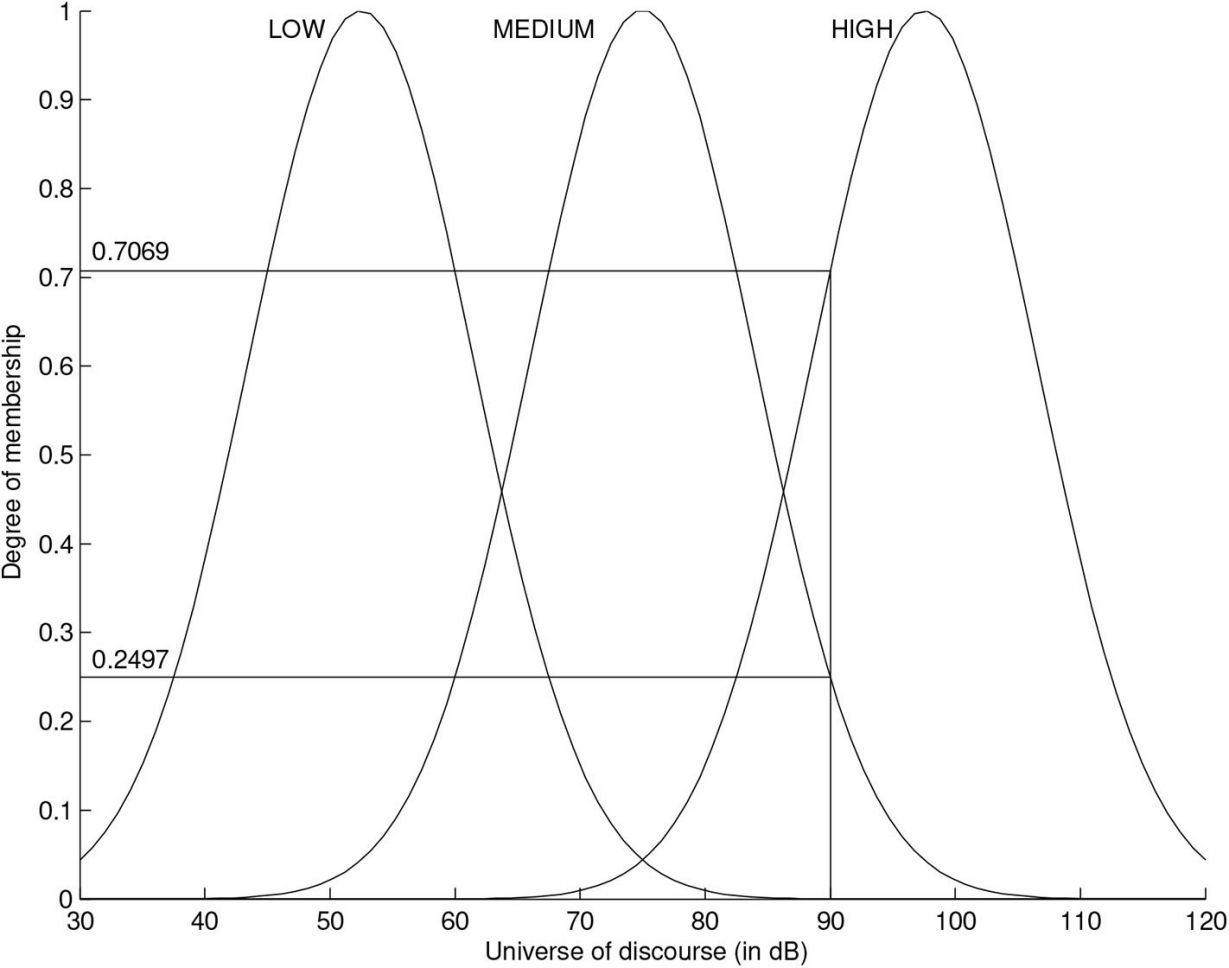
Fuzzification of the concept “intensity.” The intensity level in dB is fuzzified into three fuzzy sets, labeled LOW, MEDIUM, and HIGH. The crisp value of 90 dB belongs to both the HIGH and MEDIUM sets in different degrees.

Fuzzification and defuzzification are critical operations in fuzzy theory, as both of these operations connect the fuzzy set domain and the real value scalar domain (Roychowdhury and Pedrycz, 2001). Methods and techniques for fuzzification and defuzzification are an active line of research, and several approaches are constantly proposed in the literature. We will now illustrate one of the simplest strategies for fuzzification. Figure 1 shows the fuzzification of the physical variable “intensity” which is often associated with loudness. Employing fuzzification, a variable or concept can be classified into one or several fuzzy sets. In this particular case, there are three fuzzy sets to which “intensity” can be classified into, denoted “LOW,” “MEDIUM,” and “HIGH.” The membership functions of these fuzzy sets are Gaussians and the universe of discourse *X* contains intensities between 30 and 120 dB. In this example, a 90 dB intensity level belongs 70.69% to the fuzzy set “HIGH,” 24.97% to the fuzzy set “MEDIUM” and 0% to the fuzzy set “LOW.”

#### 2.2.2. Operations on Fuzzy Sets

Operations can be defined for fuzzy sets in the same way they are defined in traditional set theory and also in several different ways. The most important fuzzy operators and the way they are typically defined are:

- Complement. $\bar{\mu }\left(x\right)=1-\mu \left(x\right),x\in X$. The complement groups all the elements that do not reside in the set μ(*x*).
- Scalar product. μ(*x*) = *S* · μ_1_(*x*), *x* ∈ *X*. A fuzzy set can be multiplied by a scalar *S*.
- Power. $\mu \left(x\right)={\left[\mu_{1}\left(x\right)\right]}^{m},x\in X$. The power operation elevates a fuzzy set to a particular number *m*. The case *m* = 2 is known as the *concentration* of a fuzzy set.
- Union. μ_∪_(*x*) = μ_1_(*x*) ∨ μ_2_(*x*) ∨ … ∨ μ_*n*_(*x*) = *max*(μ_1_(*x*), μ_2_(*x*), …, μ_*n*_(*x*)), *x* ∈ *X*. The union of two or more fuzzy sets joins all the elements of the universe of discourse that belong in some degree to any of those sets. This operation can be done with any triangular co-norm. In this particular implementation, we unite fuzzy sets by selecting the maximum values among them.
- Intersection μ_∩_(*x*) = μ_1_(*x*) ∧ μ_2_(*x*) ∧ … ∧ μ_*n*_(*x*) = *min*(μ_1_(*x*), μ_2_(*x*), …, μ_*n*_(*x*)), *x* ∈ *X*. The intersection of two or more fuzzy sets extracts all the elements of the universe of discourse that belong in some degree to all of those sets. This operation can be done with any triangular norm. In this particular implementation, we unite fuzzy sets by selecting the minimum values among them.

In set theory, both the intersection and union operators produce one set. This is also known as aggregation. In the case of crisp logic, the only way to aggregate one or more sets is by these two operations. However, in the case of fuzzy sets, aggregation can be achieved by several averaging operations, some of which are not necessarily symmetric. For example, it would be possible to specify different weights for each fuzzy set involved (Belohlavek and Klir, 2011). This type of aggregation seeks the averaging of several fuzzy sets into one, and it is not to be confused with the process of rule aggregation, which will be discussed shortly.

### 2.3. Fuzzy Systems

Fuzzy systems are model-free estimators, they estimate input-output functions where the inputs are fuzzified and the outputs defuzzified. They estimate a function, and can approximate one with any degree of accuracy, without an underlying mathematical model relating inputs to outputs. Fuzzy systems learn from experience codified into numerical or even linguistic data (Kosko, 1992). A general fuzzy system consists of a rule base, an inference engine, and fuzzification and defuzzification stages (Klir and Yuan, 1995). It operates repeating a cycle of three steps:

1. Fuzzification. Input variables are converted into fuzzy variables.
2. Fuzzy inference engine. The fuzzified measurements are evaluated by the rule base, resulting in one or several fuzzy rules describing the universe of possible actions.
3. Defuzzification. The fuzzy outputs are converted into a single value or a vector.

#### 2.3.1. Fuzzy Rules and Inference

One of the goals of fuzzy logic is to emulate the way humans reason, which is typically just by some imprecise rules and common sense. Most of the decisions humans take can be modeled after computer-like *if-then statements*, based on expert knowledge or common sense. However, fuzzy rules can also be learned from data (Kosko, 1992). One example is the FUZZEX algorithm which can learn rules from a corpus of data mapping inputs to outputs, in the same fashion that a neural network does (Finn, 1999).

Formally, a fuzzy rule is a conditional of the form IF *X* is *A* THEN *Y* is *B*, where *A* and *B* are fuzzy sets (Kosko, 1993). Typically, fuzzy systems contain a large rule base and the method by which the computation of the contribution of each rule is achieved is known as *aggregation*. There are two main aggregation strategies, one connecting the rules with AND operators, and another where they are connected by OR directives (Ross, 2010). In the first, the aggregated output is obtained by the intersection of all the individual rules, while in the latter the output is aggregated by the union of the contribution of all rules.

The process of aggregating all the rules in parallel is called fuzzy inference. Different inference methods can be employed depending on the task in question. One of the most popular is the Mamdani method, proposed in 1975 by Mamdani and Assilian (Ross, 2010). Several variants of this method exist, for example the min-max method where a fuzzy rule would have the form:

$$\;IF\;x_{1}\text{ is }A_{1}^{k}\;AND\;x_{2}\text{ is }A_{2}^{k}\;THEN\;y^{k}\text{ is }B^{k}\text{ for }k=1,2,\ldots$$

where $A_{1}^{k}$ and $A_{2}^{k}$ are fuzzy inputs and *B*^*k*^ is the desired output. For *r* disjunctive fuzzy IF-THEN rules, the aggregated fuzzy output will be:

$$\begin{array}{l} \mu B^{k}\left(y\right)=\underset{k}{\max}\left[\min\left[\mu A_{1}^{k}\left(\text{input}\left(1\right)\right),\mu A_{2}^{k}\left(\text{input}\left(2\right)\right),\ldots \;\right]\right]\;\\ \text{for }k=1,2,\ldots ,r \end{array}$$

After inference, there comes defuzzification, a process to which several approaches exist (Ross, 2010). One of the most used ones is the centroid method, where the center of mass of the aggregated fuzzy output is computed as a scalar value.

Another widely used inference method is the TSK or Sugeno method proposed by Takagi, Sugeno, and Kang. In this method, two inputs *x, y* and one output *z* are associated by a rule of the form:

IF x is A  AND  y is B  THEN   z is z=f(x,y)

where *z* = *f*(*x, y*) is a non-fuzzy function of the inputs *x* and *y* (Ross, 2010). This inference function can be any function that describes the output of the system within the fuzzy region that the particular fuzzy rule encompasses. One advantage of the TSK method over the Mamdani strategy is that it requires less computation time by avoiding the defuzzification stage, which can be computationally challenging if the rule base is large enough.

### 2.4. Fuzzy Logic vs. Deep Learning

Both neural networks and fuzzy systems are numerical frameworks used to estimate input-output functions without an underlying mathematical model of how inputs relate to outputs. In this sense, they are model-free estimators (Kosko, 1993). Both approaches have been proven to be universal approximators for any non-linear function to any degree of accuracy (Kosko, 1994; Ying, 1998).

Neural networks excel at learning and adapting under uncertainty scenarios. It is no surprise then that deep learning has emerged as perhaps the most important branch of AI due to its unprecedented capacity of learning data in an unsupervised manner and superb results in tasks of classification and estimation. However, due to the high complexity of some network architectures and the large amount of data that is needed for training, it is very hard to understand what is being learned or even why some systems work. Indeed, a large amount of current research in deep learning seeks to understand what are networks learning. In short, neural networks can do amazing jobs at the cost of inaccessible knowledge.

Fuzzy logic, on the contrary, is all about knowledge representation. It is very clear what is being learned and represented as all knowledge is encoded in the rules of the fuzzy system. There is no major mystery as to why fuzzy systems work. And these systems can also operate under uncertainty and require almost no data, besides a couple of examples or common sense to derive the rules from.

In the case where the number of inputs to a fuzzy logic system is significantly less than the number of outputs, then the system mimics the behavior of a latent space, in the sense that its rules, which depend only on a few inputs, are a compact representation of the dynamics of the many output parameters. However, the main difference with the typical latent spaces that can be found in auto-encoders and other types of neural networks is that this fuzzy latent space is constructed based on simple rules and it is not inferred or learned from data. In other words, this is a well-understood and totally determined latent space.

Fuzzy systems offer nice opportunities for creative applications, as they are able to mimic some characteristics of human reasoning. The parallel calculation of fuzzy rules generally reduces the calculation time compared to traditional deep learning techniques or mathematical approaches. Knowledge is encoded employing fuzzy rules that can easily be specified as IF-THEN statements, with simple linguistic terms, using common sense, and they can be easily adjusted.

## 3. Fuzzy Logic for Musical Applications: A Survey

Back in 2001, Landy (2001) signaled fuzzy logic as one of the important potential domains of development for the future's music world. We believe that fuzzy logic has not yet reached its full potential in the service of musical purposes, due perhaps to the exponential growth and development of deep learning and other AI techniques. However, fuzzy logic has found its way into several domains related to audio applications and musical creative activities, as we report now.

### 3.1. Acoustics, Psychoacoustics, and Digital Audio Processing

Demichelis et al. (1983) proposed an automatic recognition method of plosive consonants, by using a fuzzy model of the human speech perception and integration mechanisms. The rules of the system were designed taking into consideration prior research done by psychologists and phoneticians in the generation and perception of these types of consonants by the human brain, which can be characterized by several acoustic cues. The authors found out that the performance of the system drastically improved when more significant cues were added to the rule base.

Civanlar and Trussel (1986) designed an audio signal restoration method based on a fuzzy system that models a priori information. The authors combined exact knowledge about the signal to be restored with partial and incomplete information. The original signal and all reasonable solutions belong to a high degree to the feasibility set of possible solutions, while rejected solutions have lower membership degrees. The measure of this set gives an approximation for the quality of the solution. This method was shown to be successful in many restoration situations where other conventional techniques to that date had failed.

Kostek (1999) designed a fuzzy controller of a pipe organ. The system links the opening procedures of the pipe valves to the manner of depressing the keyboard. The proposed solution utilizes a velocity-sensitive MIDI keyboard, connected to a computer with a special fuzzy microprocessor card and a buffer to control an array of electromagnets. These, in turn, control the pipes. Inputs to the fuzzy controller were key number and velocity. The output can belong to the following membership functions: low current, medium current, and high current. The system produced one fuzzy output, associated with the current applied to the electromagnet coils, based on eleven rules.

Breining (2001) developed a fuzzy step-gain control procedure for adaptive filters to be used in acoustic echo cancellation situations. Many step-gain estimators become unreliable under adverse environments. This fuzzy logic-based controller used a step-gain estimator combined with a double-talk detector, resulting in a highly convenient and relatively simple method compared with traditional alternatives.

Meng et al. (2002) designed an analytic method to extend the sound impulse response of a room, using knowledge from extrapolation theory for band-limited sound signals. The evaluation of the method was conducted using a fuzzy clustering algorithm. The authors use similarity perceptual judgment tasks to compare the similarity between the extrapolated real impulse responses. Typically these kinds of perceptual measurements estimate a similarity matrix with methods, such as Kruskal's multidimensional scaling. However, considering that the terms similar or dissimilar are not entirely crisp concepts, fuzzy logic was added to transform the similarity matrix into a fuzzy clustering matrix.

Malcangi (2008) constructed a fuzzy audio-pattern recognition algorithm targeted for usage in very low-cost embedded systems, to automate human-machine interaction. This system was built on top of feature extraction algorithms and a rule base constructed by a self-learning process. The fuzzy logic recognition engine used membership functions according to the spectrum of a particular audio frame to be recognized. In consequence, if the audio pattern was, for example, a vocal utterance, then the membership function would be modeled according to the spectrum envelope of the stationary components of the speech. In such a manner, a set of membership functions covering all the stationary speech sounds to be recognized must be generated. By using a different dictionary of membership functions, other kinds of acoustic signals can be recognized by such a system.

Gonzalez-Inostroza et al. (2015) proposed a fuzzy-logic based equalizer for musical genres, by incorporating significant audio descriptors that allow for the recognition and description of diverse musical genres. These descriptors feed a fuzzy logic inference system, whose outputs are the required equalization levels for each frequency band. The rules of the system were derived from the analysis of a well-known music database encompassing ten different musical genres. Their approach works for songs that exhibit multiple genre characteristics, that are difficult to classify into one category, or that mix genres.

### 3.2. Music Listening, Emotion, and Analysis

Milicevic (1999) aimed to aid composers to create more appealing music for a wider public. Assuming that composers usually seek a positive cultural response to their music, the authors built a fuzzy adaptive and emotion-based music system that can reduce the internal fuzzy entropy of the compositions, making them more appealing to people and able to produce positive emotional responses while listening to it. They tried their system in the special case of computer music.

Friberg (2005) was able to use a fuzzy system for the analysis of the emotional expression and body movements in musical performances in real-time. Parameters, such as articulation, tempo, intensity, and motion descriptors were used as inputs to a fuzzy mapper able to translate these variables into one of three possible outputs: happiness, sadness, and anger, all related to emotion. The rule base was constructed considering qualitative data from former studies.

Yang et al. (2006) also developed a music emotion classification system based on fuzzy logic. They declare that “due to the subjective nature of human perception, classification of the emotion of music is a challenging problem,” as feeling and emotion states provoked by music could be unequal to different people. Their approach estimates the likelihood that a given segment extracted from a song belongs to a particular category of emotion. Their system can measure emotional strength to track the variation of different musical emotions provoked by a song.

Maristany et al. (2016) have done soundscape quality analysis by means of fuzzy logic. They conducted a comparative analysis, based on surveys and psycho-acoustic estimations, in open locations around the city of Córdoba in Argentina. They found out that there is a non-linear relationship between these indicators and the audible qualities of the spaces. Fuzzy logic emerged then as a suitable tool to model this non-linearity, confirmed by the model performance and the perceptual outcomes from the users. The authors found that this approach can be applied not only to soundscapes, but in other studies where perception must be confronted with objective measurements.

Hasanzadeh et al. (2019) constructed a fuzzy cascade model designed to predict the emotional content of pieces of music using electroencephalographic (EEG) signals. Users listened to musical excerpts while their emotional appraisal response was estimated as a value along two emotional axes (valence and arousal). The proposed fuzzy model consists of parallel cascades with each cascade containing a single multi-input/single-output fuzzy logic-based system. The authors compared this approach to several alternative methods, including recurrent neural networks, and concluded that the fuzzy approach exhibited the best performance.

Kasinathan et al. (2019) developed a music recommendation system based on a fuzzy inference engine that considers user activities and emotion as part of the recommendation parameters. The authors describe that their fuzzy inference system can decide on music recommendations based on the user's music listening habits as well as expert knowledge about music genres and their effects on humans. The user's preference data is fed into the fuzzy system to obtain a decision that returns a score corresponding to the recommended music track. The top ten music tracks with the highest recommendation score are provided as recommendations for the user.

### 3.3. Music Information Retrieval and Performance

Orio and Pirro (1998) coded gestures made during interactive musical performances in real-time by a neuro-fuzzy system. One of the basic contributions of their work is using only two different levels for the codification of human gestures. These levels usually carry a significant amount of information about the performer's intentions. But additional information is carried by nuances of the gesture, and these can also be analyzed, capturing the detailed performance of each gesture. This two-level approach was applied in a system for interactive piano performances. Nuances were analyzed in terms of linguistic labels. This is where fuzzy logic plays an important role, given its suitability to handle semantic expressions. Depending on the kind of desired performative nuances, fuzzy controllers were developed. Loudness and tempo are used as inputs, and the system then calculates the level of, for example, “urgency” of the musician. This information is later used by the system to musically respond in real-time to the human performer.

Usa and Mochida (1998) used a fuzzy system in the simulation of the gestures of an orchestra conductor. The system is capable of recognizing some of the most common conducting elements of conductors. In particular, the beat recognition system was built on top of a fuzzy model of actual orchestra musicians' recognition.

Liu and Huang (1998) implemented a system that discriminates news reports from broadcast ads or music in news programs based on the information contained in the audio signals. Four features were extracted from the audio data. Both a simple threshold and a fuzzy classifier were implemented to classify the audio data. In the case of the fuzzy classifier, descriptors were associated with fuzzy sets and the influence of each feature was combined to obtain the final classification decision. Results reported an improvement using the fuzzy classifier compared to the threshold-based system.

Weyde and Dalinghaus (2001) recognized rhythmic patterns with a neuro-fuzzy system, which determined grouping and group relations between two sequences (comparison) or within one sequence (analysis). The system makes use of knowledge and learning from data and it is open for the integration of different features and rules. The system defined by the rules can be trained to prefer certain interpretations over others by example.

Liu et al. (2002) propose a fuzzy system designed to classify and retrieve audio clips, inspired by the fuzzy nature of human perception. Various extracted features were used as input to a fuzzy system, whose outputs belonged to two types of classes. The rule base was constructed from characteristics extracted from the clips. The results show that the system can discriminate between speech and music and that it can be extended for the classification of more types of audio clips.

Monti and Sandler (2002) developed a system able to translate audio directly into MIDI data. The system contains a fuzzy inference system that achieves polyphonic note recognition as a part of the overall process. First, spectral peaks from a spectrogram are selected by the algorithm. Harmonically related peaks are grouped into note candidates. If a note candidate receives a good rating, it is transformed into a note hypothesis. Finally, the hypotheses that survived an activation time threshold become active notes. The fuzzy inference system takes the spectral peaks that were not selected into the note continuation process and creates new candidates. The new candidates are then evaluated by the inference system to become note hypotheses. The membership functions used in this system classify notes into low, middle, and high and take into consideration pitch, harmonic rate, and relative energy.

Leon and Liern (2010) modeled musical notes and tuning systems as fuzzy entities to integrate tuning theory and musical practice. The authors were able to combine different tuning systems into a simpler fuzzy model that reflects both the idea of proximity between different notes and whether their configuration, in terms of a specific tuning system, is sufficiently similar for practical musical purposes.

Knudsen et al. (2019) propose that “to collaborate and co-create with humans, an AI system must be capable of both reactive and anticipatory behavior.” With this objective in mind, the authors considered a mixed human-robot duo, more precisely a piano and a virtual robotic drummer, and they designed a fuzzy logic-based system to determine the performance features of the drummer as a function of what the human pianist performs. While the system exhibited only limited anticipatory capabilities, the behavior of the drummer was judged to be satisfactory by musicians in initial evaluation experiments.

### 3.4. Musical Composition and Generation

Lee and Wessel (1993, 1995) were among the first researchers to incorporate a fuzzy reasoning system into the MAX real-time music programming language. They labeled their system MaxFUZ, and it implemented fuzzy variables, sets (limited to trapezoidal shapes), and both Mamdani and Sugeno rules. This was the first interactive fuzzy system that worked in real-time inside MAX to our knowledge.

Almost at the same time, Elsea (1995) utilized fuzzy logic features to tackle problems in the analysis and composition of music. In his work, pitches and dynamics were represented as fuzzy sets and fuzzy reasoning is used to produce chord inversions and sequence of chords. These ideas were implemented in software as external objects for Max/MSP, called L-objects, which provide fuzzy operations and manipulation of fuzzy sets.

Kiseliova et al. (2005) developed an interpretation fuzzy algorithm, based on top of a rule base designed by an experienced pianist. Their approach relies on both conventional and more advanced information decision strategies. This system, given a known piece of music, creates a MIDI-based interpretation of the piece. Their general objective is to transform a mechanical performance of a piece of music into a much more human-like interpretation by applying the knowledge of an expert performer in the form of a fuzzy rule base.

Cádiz (2004, 2006a) proposed a fuzzy logic system to convert visual information into sonic information and vice-versa. This model is useful to generate audiovisual content, given either the visual or sonic content in advance. Parameters in one domain are fuzzified and fed into a fuzzy inference engine that generates parameters in the other domain. This fuzzy mapping is inspired by the ideas of isomorphism and synaesthesia. Isomorphism determines whether two different modalities can be mapped onto each other based on the fact that perturbations into one of them consistently cause changes in the other, while synaesthesia occurs when a stimulation in one sensory modality automatically provokes a perceptual outcome in a secondary sensory modality when there is no direct stimulation to it.

Yilmaz and Telatar (2009) identified key areas where fuzzy logic can be used for the composition and generation of music: harmonization, orchestration, improvization, and composition. They propose to focus on the harmonization with constraints as a way of tackling these three areas. In particular, they proposed a fuzzy feedback decision system designed to perform accompanying tone generation dynamically. They applied this system to the particular problem of note-against-note two-voice counterpoint (Yilmaz and Telatar, 2010). Their method considers membership functions and rules that mimic some known rules of music theory, and their implementations provide feasible procedures when compared to those of established music theory.

Suiter (2010b) devised a conceptual framework for composing expressive music based on fuzzy logic, aimed toward reducing the number of musical decisions that a composer must make at the micro-level and focusing on those that contribute to expressiveness the most. A fuzzy system is used to trace the trajectory of all musical details of a composition, encompassing each element and their combinations.

López-Ortega and López-Popa (2012) developed a two-dimensional recursive fuzzy method assisting composition for MIDI-based musical works based on fractal structures. In their approach, notes evolve according to a particular fractal trajectory. Tempos and duration can remain fixed or they also can follow the fractal structure. Additionally, the set of produced pitches are translated into tones belonging to a previously determined musical scale.

Kuo et al. (2015) created a real-time emotion-based music accompaniment system through a fuzzy logic tempo controller, and an additional genetic evolutionary melody generation system. Harmonic chord progressions were generated using known music theory rules. For the fuzzy tempo controller, they used a range of 60 to 180 beats per measure, and the fuzzy output is used to adjust the current tempo compared to a target one.

Lucas et al. (2017) developed a method for representing human emotions in the context of human-machine musical composition based on fuzzy logic. A knowledge base of human-produced melodies and human-labeled emotions associated with them, in the form of a Markov chain process, is used to generate new melody patterns, which are later classified into emotions by a fuzzy classifier. These new melodies can be of later use to compose music with specific emotional targets in mind.

Guliyev and Memmedova (2019) modeled some compositional decisions as the requirement to construct relationships between controllable elements in music, in particular pitch, duration or amplitude, and a consequent evaluation. They established these relationships as sets of IF…THEN fuzzy rules with antecedents, the input parameters, and a consequent, the evaluation of the generated musical output. This method can be thought of as a “preference ordering” of the attributes of a particular piece of music.

### 3.5. Sound Synthesis

Miranda and Junior (2005) introduced a novel Markov fuzzy model for granular synthesis. While Markov chains control the temporal evolution of the sound, their fuzzy system defines the granular structure of the sound. In this sense, this method extends the idea of a grain into the concept of a fuzzy grain. A fuzzy grain contains several fuzzy parameters: frequency, amplitude, and membership values of each Fourier partial of the grain, or in other words, its weighted harmonic content.

Schatter et al. (2005) proposed a graphical user interface for the generation of electronic sounds with a synthesizer. Twenty-three aural parameters were reduced to five parameters controlling the visual metaphor utilizing a fuzzy logic controller. The system allows knowledge-based mappings that are adaptable to each user. There are two modes of operation: in the manual operation mode, the system is used to record the parameter-input of the user, followed by the generation of fuzzy controllers. In the automatic mode, the system has to find parameter combinations, employing genetic algorithms.

Cádiz (2006b) has proposed an approach for the compositional control of computer music based on fuzzy logic. In this case, the control of the compositional process derives from the fuzzification of the synthesis parameters of interest, while the rule base can be specified at will by the composer, according to his objectives. The author provides five different applications of this type of synthesis control in the context of spectral synthesis, physical modeling, granular synthesis, particle-based synthesis, and audiovisual composition, exemplifying a significant number of situations in which such an approach gives suitable results.

Lucas and Pelaez (2019) implemented a granular synthesis method based on harmonic rules and fuzzy logic. In this method, each grain is positioned in a two-dimensional space arranged in the same fashion as the circle of fifths. A fuzzy logic prioritization algorithm is used to order the grains in the vicinity of a particular performing area inside this two-dimensional space. The algorithm takes frequency and energy levels of each grain in the vicinity as inputs and produces a prioritization index as a result.

## 4. The Fuzzy Logic Control Toolkit

The Fuzzy Logic Control Toolkit (FLCTK) (Cádiz and Kendall, 2006; Cádiz and Gonzalez-Inostroza, 2018) is a collection of software tools implemented in MaxMSP^1^, a sound synthesis environment that allows for the design and usage of a generic fuzzy inference system in real-time. An important feature of this software is its capability to import and export fuzzy systems in the fis file format, a popular fuzzy logic specification used by MATLAB's Fuzzy Logic Toolbox^2^. This common shared file format allows a user to design and troubleshoot a complete fuzzy system in MATLAB, export it as a fis file, and then import the same system into the FLCTK or vice-versa.

**Figure 2:**
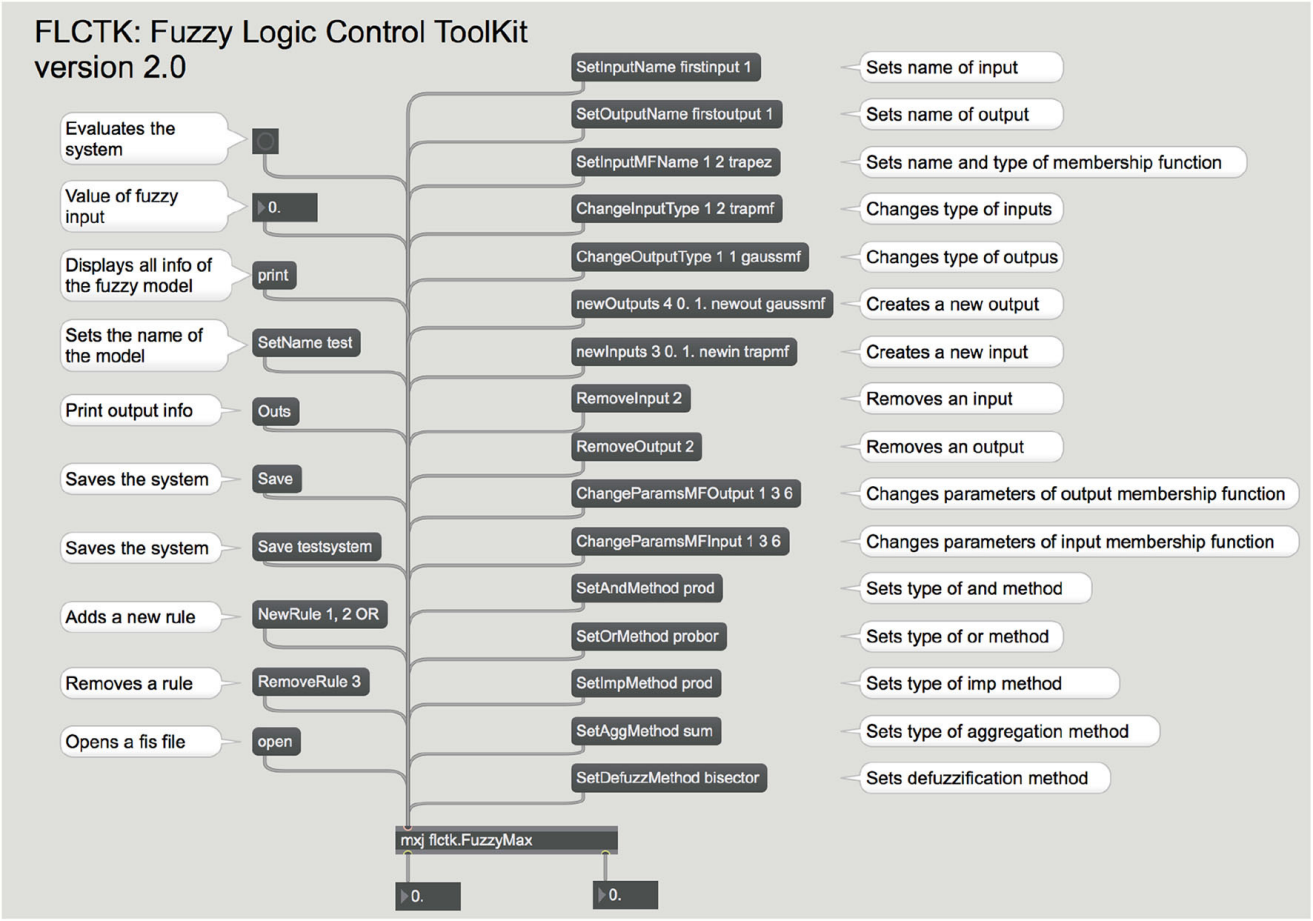
Help screen of the Fuzzy Logic Control Toolkit (FLCTK) in the MaxMSP environment. Inputs, outputs and rules can be added and removed on-the-fly.

Figure 2 displays a screen-shot of the MaxMSP help patch of the FLCTK's external flctk.Fuzzy with all its options. This external object can load a fuzzy system or create one on-the-fly by sending messages to it. In this example, details of the fuzzy system in use are displayed in the Max window. Some messages can select specifics for implication, aggregation, fuzzification and defuzzification, number of rules, their weights, and whether AND or OR operators should be used. All fuzzy inputs and outputs can be defined with either triangular or Gaussian membership functions, and labels can be created for each of them.

This toolkit is very simple to use inside MaxMSP and a complete fuzzy system can be designed by sending the appropriate messages to the flctk.Fuzzy object. This object can be created based on an existing fis file designed off-line in MATLAB. In this case, the path to the fis file should be specified. Another option would be to initialize the object with several inputs and outputs, in which case the external will create all necessary fuzzy variables with a standard configuration using Gaussian membership functions and inputs and outputs consisting of five fuzzy sets each. This configuration can be altered after the system was created by sending modifier messages. Once inputs and outputs are created, rules can be added one by one by sending a message specifying the inputs and outputs involved in each rule, the aggregation method, and specific weight for each rule. Rules can be deleted and tested on the fly, to customize the system's behavior.

The FLCTK can be downloaded from its github website at https://flctk.github.io/. The package contains the source Java code, compiled code, help files and video examples, some of which are detailed below. Also, a standalone version, written from scratch in C++ and based on the Open Sound Control protocol (Wright et al., 2003) is in the works at the time this article was published.

## 5. Examples

We now provide four examples, developed by the author using the FLCTK, that illustrate the power of fuzzy logic for audio and music generation, in the specific domains of computer music and algorithmic composition, sound synthesis, and parametric control. These examples are purposely very simple, as they were designed to clearly show the effect of fuzzy logic when applied to very basic ideas. Illustrating videos of each of the examples can be found in the Supplementary Material.

**Figure 3:**
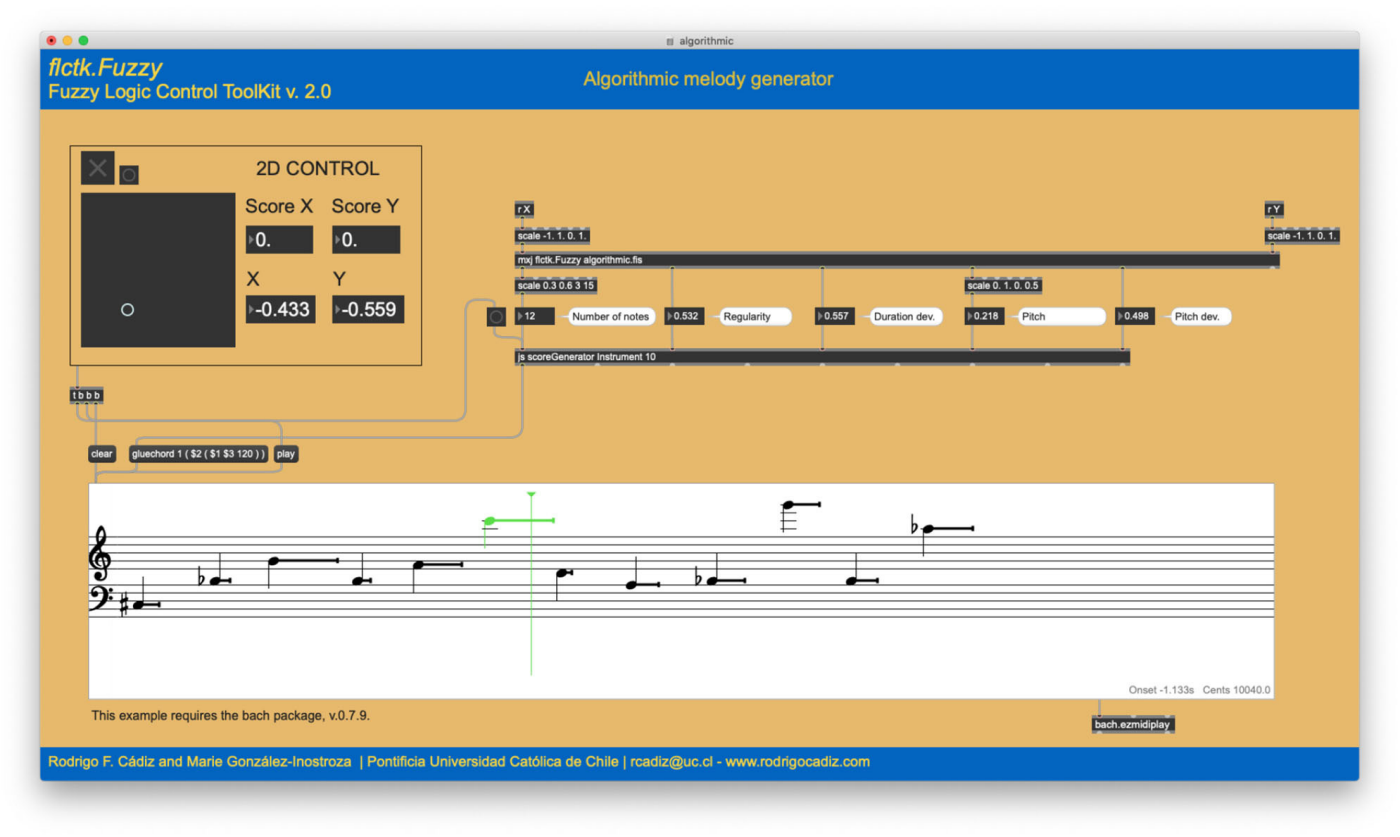
Algorithmic melody generator example 1. The 2D coordinates [−0.433, −0.559] of the latent space generate a 12-note melody with a large range of pitch variations.

**Figure 4:**
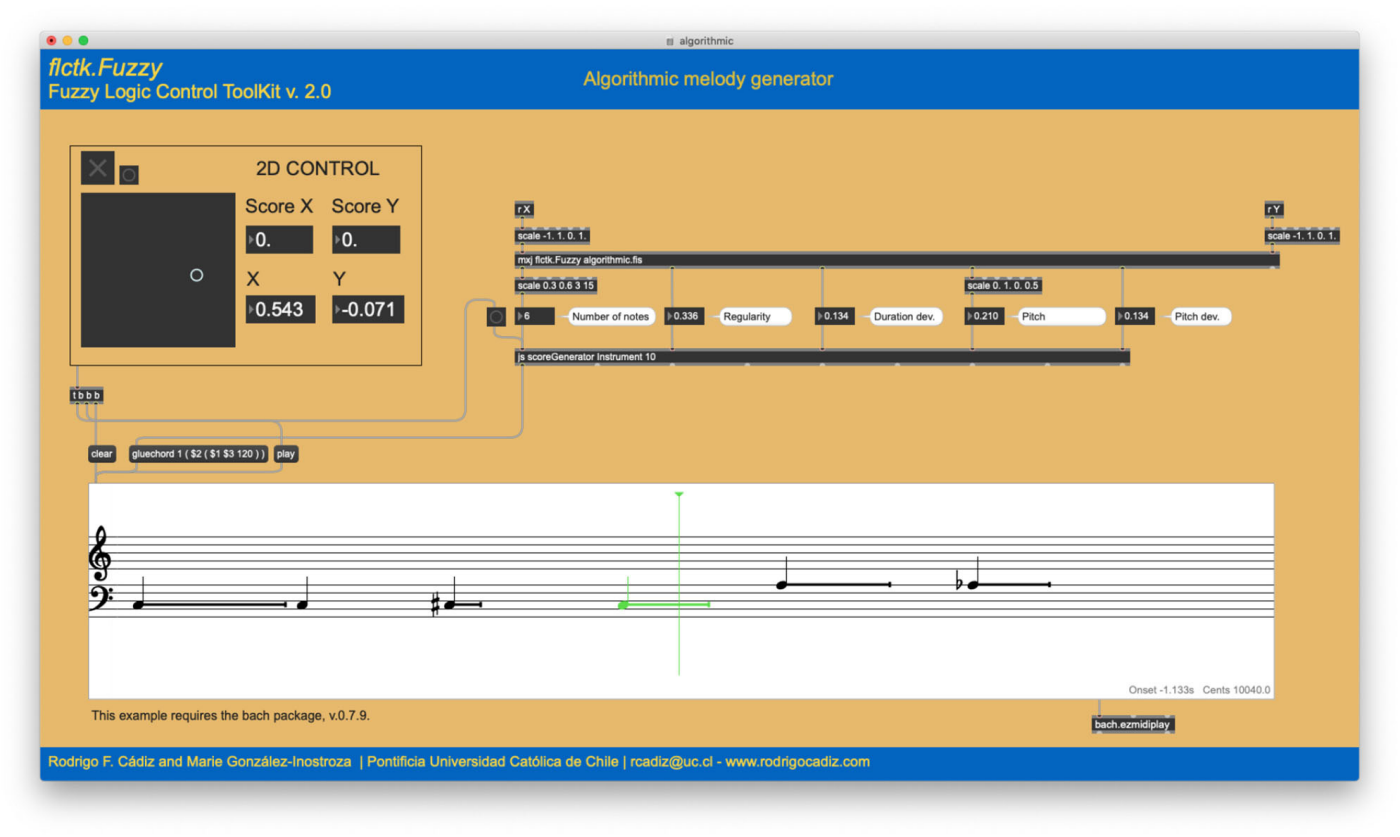
Algorithmic melody generator example 2. The 2D coordinates [0.543, −0.071] of the latent space generate a six-note melody with a rather small range of pitch variation in the lower register.

For the algorithmic composition and parametric control examples, we utilize a bi-dimensional controller (shown in Figures 3, 4, **8**) as a very simple control interface. The bi-dimensional controller has a square shape and a pointer (small circle) that tracks the coordinates of the mouse as the user moves it. Both axes have a range of 2.0 (from −1.0 to +1.0). The origin (0,0) is located at the center of the square. The controller also accepts pointer coordinates via internal messaging. In this way, the controller can in turn be controlled not only by the mouse but by any kind of two-dimensional process. In the following examples, the coordinates of the controller are fed into custom fuzzy systems designed for each particular case. As these fuzzy systems contain more than two outputs, this controller behaves as a latent space, which is a compact representation of the high-dimensional output parameter space.

### 5.1. Algorithmic Composition

Algorithmic composition is simply the use of algorithms to compose music. This is a very common practice in the history of music, as “for centuries musicians have been proposing methods that can be considered as algorithmic in some sense, even if human creativity plays a key role” (Fernández and Vico, 2013). There is a great variety of algorithms that have been proposed for music composition, including simple recursive equations, chaotic systems, re-writing systems, and many others (Nierhaus, 2009; Edwards, 2011).

Algorithmic composition using fuzzy logic is proposed here as another alternative. Fuzzy logic, as we have previously discussed, is flexible enough to be applied to many different composition-related contexts and situations. This particular example consists of the generation of a very simple melody, where the number of notes, their pitch range, and their duration are determined by a fuzzy inference system. The inputs to the system are the two outputs of the aforementioned bi-dimensional controller. The outputs are the number of notes, a regularity factor, a duration deviation factor, pitch, and a pitch deviation factor, as specified by Table 1. Each one of these variables can belong to three fuzzy sets: LOW, MEDIUM, or HIGH. As there are fewer inputs than outputs, the input space is a latent space of the output space.

**Table 1:** Input and output variables for algorithmic composition example.

**Input variables**	**Output variables**
X	Number of notes
Y	Regularity
	Duration deviation
	Pitch
	Pitch deviation

*Each one of these variables can belong to three fuzzy sets: LOW, MEDIUM, or HIGH. As the number of inputs is lower than the number of outputs, the input space is a latent space of the output space*.

This system contains nine fuzzy rules, detailed in Table 2. All rules have the same weight and are connected by AND operators. A “–” indicates that the value of the fuzzy variable can be anything. As shown in Figure 3, given the coordinates [−0.433, −0.559] of the latent space, the system generates a twelve-note melody with a large range of pitch variations. Another point in the latent space will produce a different output, as is displayed in Figure 4, where the coordinates [0.543, −0.071] of the latent space output a six-note melody with a rather small range of pitch variation in the lower register.

**Table 2:** Fuzzy rules for the algorithmic composition example.

	**Inputs**		**Outputs**				
**Rule**	**X**	**Y**	**Num. of notes**	**Regularity**	**Duration dev**.	**Pitch**	**Pitch dev**.
1	LOW	–	HIGH	MEDIUM	LOW	HIGH	MEDIUM
2	MEDIUM	–	HIGH	MEDIUM	–	LOW	LOW
3	HIGH	–	LOW	LOW	LOW	HIGH	LOW
4	–	LOW	MEDIUM	HIGH	HIGH	–	HIGH
5	–	MEDIUM	LOW	LOW	LOW	LOW	LOW
6	–	HIGH	LOW	LOW	HIGH	MEDIUM	HIGH
7	LOW	HIGH	LOW	MEDIUM	LOW	HIGH	MEDIUM
8	MEDIUM	HIGH	HIGH	MEDIUM	MEDIUM	LOW	HIGH
9	HIGH	HIGH	HIGH	HIGH	HIGH	HIGH	HIGH

*All rules have the same weight and are connected by AND operators. A “–” indicates that the value of the fuzzy variable can be anything*.

### 5.2. Sound Synthesis

An audio synthesis technique, based on fuzzy logic and the idea of sound particles, is presented as a second example of the application of fuzzy logic for music generation. This technique has been shown to generate complex synthesis parametric trajectories by very simple means (Cádiz and Kendall, 2005). This example consist of a single sound particle (a sinusoidal oscillator) that possesses several fuzzy properties, labeled as time, frequency and intensity. These properties are fed into a fuzzy system that determines the temporal evolution of the particle. Each one of the fuzzy properties consists on several fuzzy sets or membership functions. Table 3 displays all the inputs and outputs used in the example. Note that as time is included as an input, complex time-dependent behaviors or trajectories can be generated.

**Table 3:** Input and output variables for the single particle sound synthesis example.

**Input variables**	**Output variables**
Time	Δ Frequency
Frequency	Δ Intensity
Intensity	

**Figure 5:**
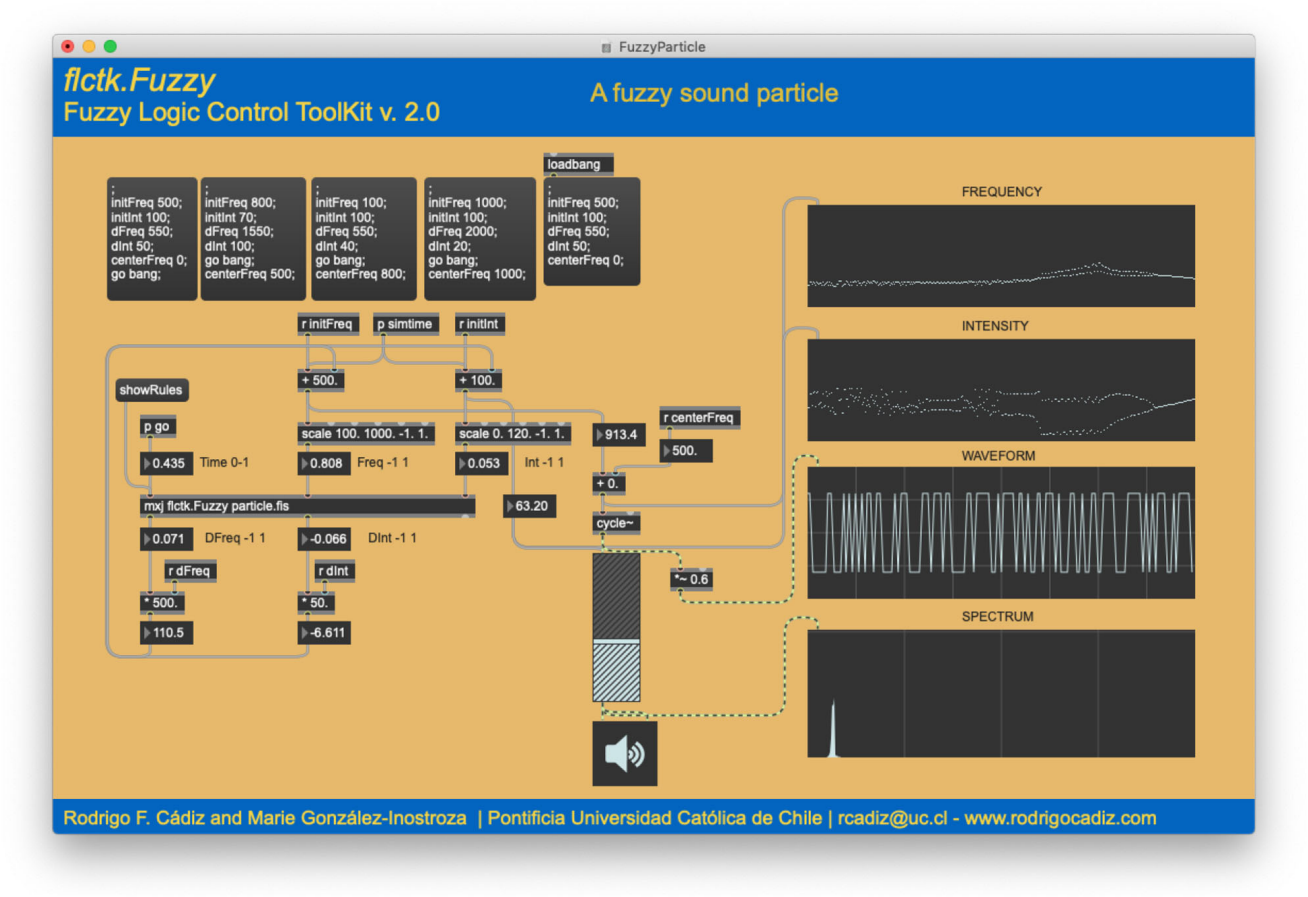
Particle sound synthesis example. In this case the fuzzy system is dependent on time. As time progresses in a linear fashion, the output variables frequency and intensity exhibit a highly non-linear behavior.

The time input variable can belong to seven fuzzy sets, labeled VERY SHORT, SHORT, MEDIUM SHORT, MEDIUM, MEDIUM LONG, LONG, and VERY LONG fuzzy sets. The frequency and intensity variables can belong to five sets: VERY LOW, LOW, MEDIUM, HIGH, or VERY HIGH. The outputs of the system are a change in both frequency and intensity. This means that, in this case, the fuzzy system is a closed-loop system, a very common design for automatic control applications. The outputs at each time step are used to recalculate the current frequency and intensity of the particle. The fifteen rules of this system are shown in Table 4. All rules have the same weight and are connected by AND operators. A “–” indicates that the value of the fuzzy variable can be anything. It is important to recall that in this example the fuzzy system is dependent on time. As time progresses linearly, the output variables frequency and intensity exhibit a highly non-linear behavior, as it can be observed in Figure 5.

**Table 4:** Fuzzy rules for the single particle sound synthesis example.

	**Inputs**			**Outputs**	
**Rule**	**Time**	**Frequency**	**Intensity**	**Δ frequency**	**Δ intensity**
1	VERY SHORT	–	–	VERY LOW	VERY LOW
2	VERY LONG	–	–	VERY HIGH	VERY HIGH
3	–	MEDIUM	MEDIUM	MEDIUM	MEDIUM
4	–	LOW	–	HIGH	MEDIUM
5	–	HIGH	–	LOW	MEDIUM
6	–	–	LOW	MEDIUM	HIGH
7	–	–	HIGH	MEDIUM	LOW
8	–	MEDIUM	–	VERY LOW	VERY HIGH
9	–	–	MEDIUM	VERY HIGH	VERY LOW
10	SHORT	–	–	LOW	LOW
11	MEDIUM SHORT	–	–	HIGH	LOW
12	MEDIUM	–	–	LOW	HIGH
13	MEDIUM LONG	–	–	MEDIUM	MEDIUM
14	LONG	–	–	VERY HIGH	VERY HIGH
15	VERY LONG	VERY LOW	–	LOW	–

*All rules have the same weight and are connected by AND operators. A “–” indicates that the value of the fuzzy variable can be anything*.

**Figure 6:**
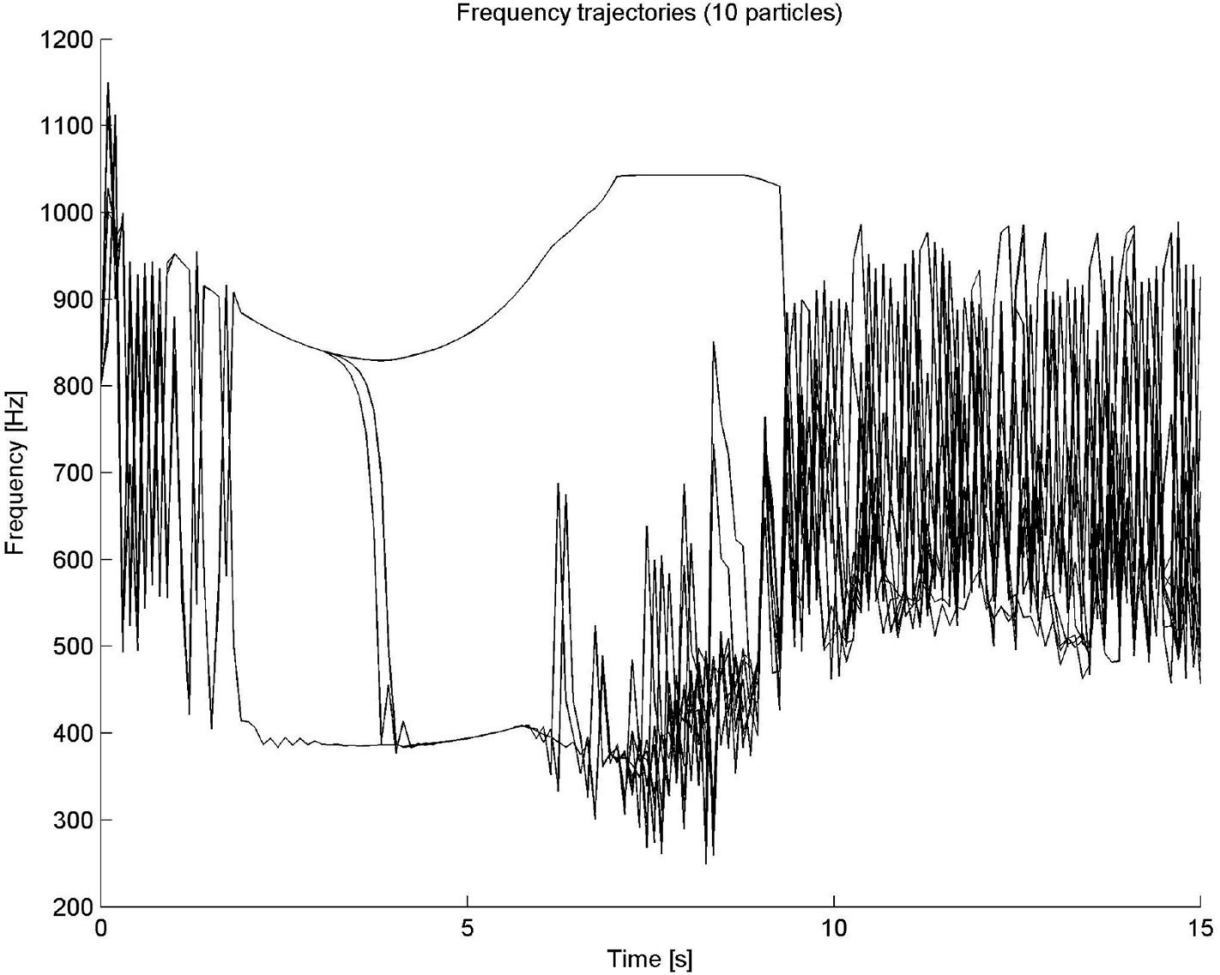
Frequency trajectories in time for a 10-particle system. As it can be seen, highly complex behavior can be generated with a few simple if-then rules.

**Figure 7:**
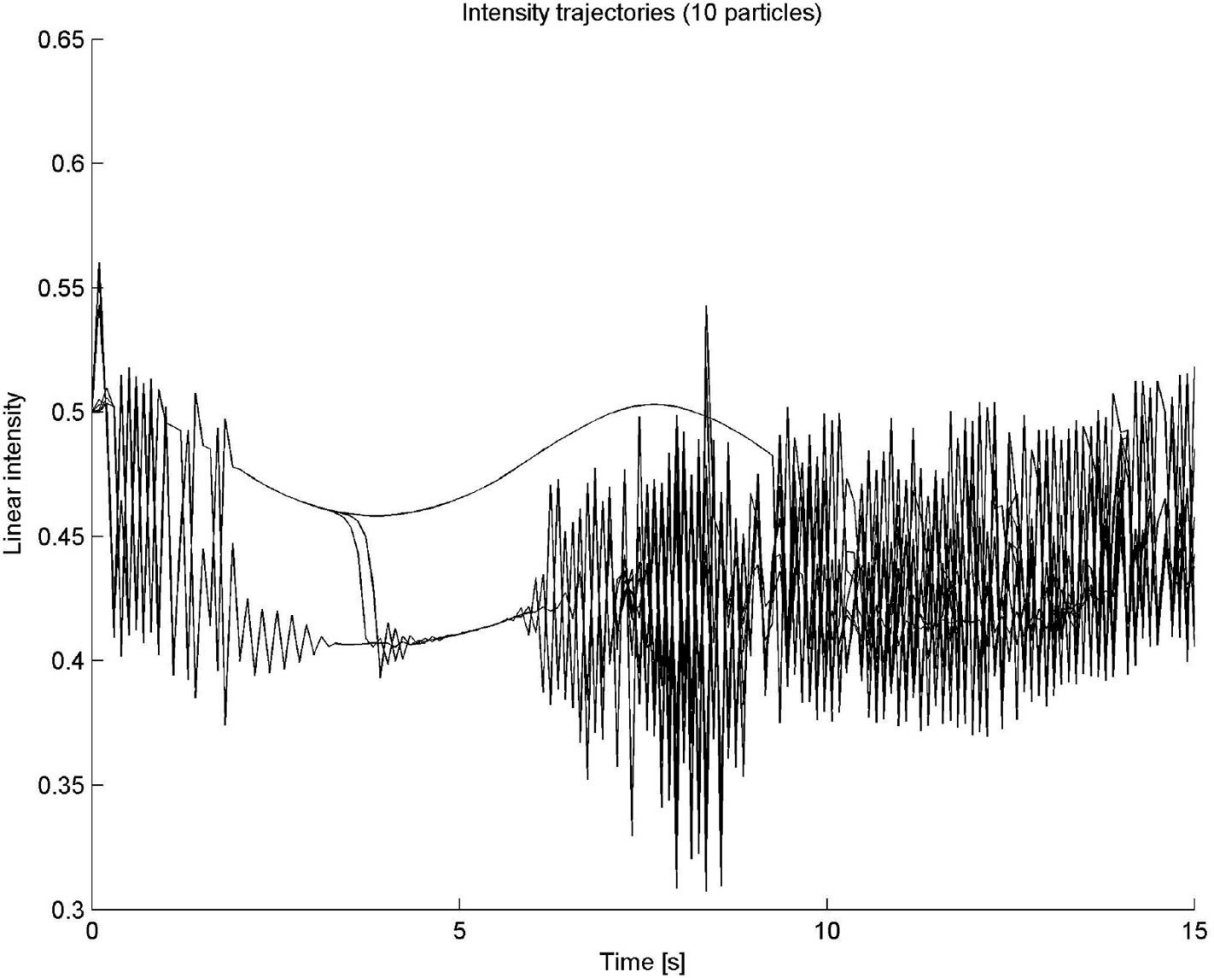
Intensity trajectories in time for a 10-particle system. As it can be seen, highly complex behavior can be generated with a few simple if-then rules.

This single particle model has been extended to many particles, as described in Cádiz and Kendall (2005). In the many particle case, two additional fuzzy properties were added: spatial position and charge. Figures 6, 7 show the frequency and intensity trajectories for a ten-particle system. In the figures, all particles shared the same initial conditions, except for random charges. The trajectories displayed in the figures are quite complex, with very different behaviors as time progresses. Sometimes they behave very chaotically and some other times, in this example most notably in the first 6 s, they follow smooth and apparently non-chaotic but rather well-defined trajectories. Some clustered groups can also be noticed. This kind of behavior is a consequence of the easiness of fuzzy logic to approximate non-linear dynamical systems.

### 5.3. Parametric Control

Granular synthesis (Dodge and Jerse, 1997) is inspired by the idea of sound particles or grains, similar in spirit to photons or particles of light. Iannis Xenakis in 1971 and Curtis Roads in 1978 were among the first to suggest granular synthesis as a viable computer music technique for producing complex sounds. This technique generates a high density of very short acoustic events or grains, resembling clouds, with a duration between 10 and 50 ms (Roads, 2004). These grain clouds typically range from several hundred to several thousand events per second. If sinusoidal functions or any pure synthesis methods are used to produce the grains, the technique is called granular synthesis, while if pre-recorded sounds constitute the grain material, people often call that granular processing. This technique often requires the user to control multiple parameters without any clear relation to what they are hearing (Wolek, 2005). This is a situation where a fuzzy logic-based control strategy could be useful.

This example consists on the control of a granulator, whose parameters are determined by a fuzzy inference system. In this specific case, granular synthesis is achieved using the nw.grainpulse object, written for Max/MSP by Wolek (2002). This object has five parameters to be controlled. The inputs to the system are the two outputs of the bi-dimensional controller used for the algorithmic composition example. The outputs are the pulse interval, buffer offset, duration, sample increment, and gain multiplier, as specified by Table 5. Each one of these variables can belong to three fuzzy sets: LOW, MEDIUM, or HIGH. As there are more outputs than inputs, the input space is a latent space of the output space, as in the algorithmic composition example.

**Table 5:** Input and output variables for parametric control of granular synthesis example.

**Input variables**	**Output variables**
X	Pulse interval
Y	Buffer offset
	Duration
	Sample increment
	Gain multiplier

*Each one of these variables can belong to three fuzzy sets: LOW, MEDIUM, or HIGH. As the number of inputs is lower than the number of outputs, the input space is a latent space of the output space*.

**Figure 8:**
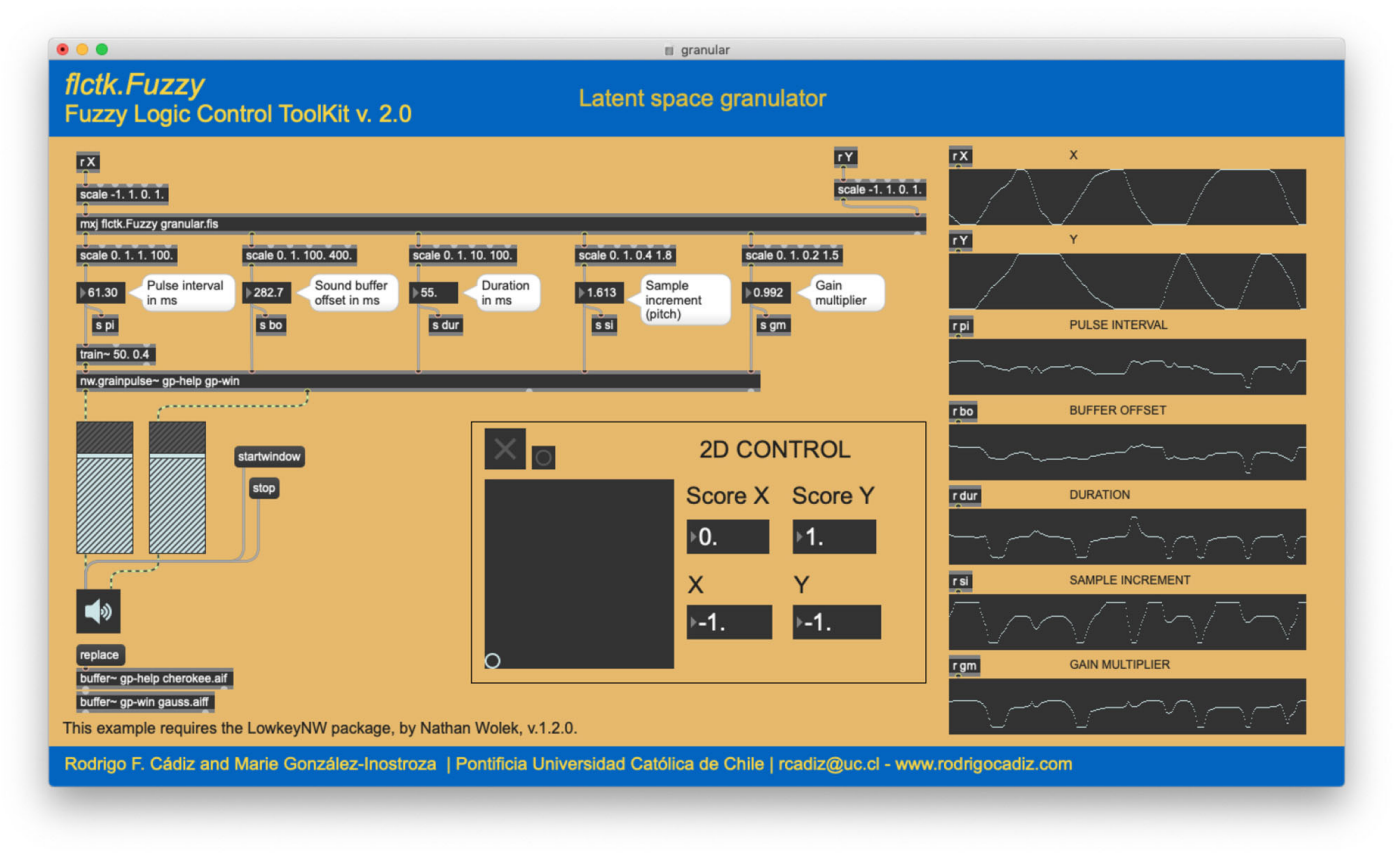
Latent space granulator example. Inputs and outputs of the fuzzy system are displayed on the right. As the latent space is explored in both the *X* and *Y* directions, the output variables exhibit different non-linear behavior. This allows the parametric control of five synthesis parameters with only two abstract parameters.

This system contains nine fuzzy rules, detailed in Table 6. All rules have the same weight and are connected by AND operators. A “–” indicates that the value of the fuzzy variable can be anything. Inputs and outputs of the fuzzy system are displayed on the right of Figure 8. As the latent space is explored in both the *X* and *Y* directions, the output variables exhibit different non-linear behavior. This allows the parametric control of five synthesis parameters with only two abstract parameters. The proposed fuzzy system effectively acts as a latent space generative model, one that can translate points from a two-dimensional parameter space into a five-dimensional space that acts directly on the sonic output, according to the nine rules of the system.

**Table 6:** Fuzzy rules used in the granular synthesis example.

	**Inputs**		**Outputs**				
**Rule**	**X**	**Y**	**Pulse interval**	**Buffer offset**	**Duration**	**Sample inc**.	**Gain mult**.
1	LOW	–	HIGH	MEDIUM	LOW	HIGH	MEDIUM
2	MEDIUM	–	HIGH	MEDIUM	–	LOW	LOW
3	HIGH	–	LOW	LOW	LOW	HIGH	LOW
4	–	LOW	MEDIUM	HIGH	HIGH	–	HIGH
5	–	MEDIUM	LOW	LOW	LOW	LOW	LOW
6	–	HIGH	LOW	LOW	HIGH	MEDIUM	HIGH
7	LOW	HIGH	LOW	MEDIUM	LOW	HIGH	MEDIUM
8	MEDIUM	HIGH	HIGH	MEDIUM	MEDIUM	LOW	HIGH
9	HIGH	HIGH	HIGH	HIGH	HIGH	HIGH	HIGH

*All rules have the same weight and are connected by AND operators. A “–” indicates that the value of the fuzzy variable can be anything*.

### 5.4. Many-to-Many Parametric Control

In computer music, sometimes the act of composing cannot be separated from the control of the synthesis process (Cádiz, 2006b). As a consequence, the compositional process can be strongly shaped by the nature of the synthesis technique that is being used. Gerhard and Hepting (2004) propose to think of composition as an exploration of a multidimensional parameter space, where a particular configuration of parameters can be represented as a point in that space. The parameters are initially de-contextualized, meaning that they only offer a possible set of future musical ideas, and compositions often means to map or re-map these parameters until targets or musical constraints are satisfied. This parameter-based approach to composition allows the composer to explore a high dimensional space of musical possibilities and essentially pick trajectories in that space that are aesthetically relevant. Dahlstedt (2001) and Gerhard and Hepting (2004) have proposed several options for this composition strategy.

As these parameter spaces become larger, more specialized tools are needed. In particular, supervised neural network methods have been often used to generate a model of the mapping from controller inputs to synthesis outputs, using training datasets consisting of examples of input/output pairs (Fiebrink et al., 2009). These kinds of networks can learn a continuous function mapping, no matter how many dimensions are involved. For these reasons, Fiebrink and Cook (2010) developed the Wekinator, a free and cross-platform open-source software application that supports interactive design and application of real-time supervised learning systems for many-to-many parametric gestural control of music.

Since version 2.0 of the FLCTK this kind of many-to-many control can also be achieved with fuzzy logic in real-time. As inputs, outputs and rules can be added on-the-fly, high dimensional parametric gestural control can be achieved with a regular fuzzy system. Please see the example video in the Supplementary Material for a better understanding of this on-the-fly mode. In the video, rules are added in real-time to map five inputs into four outputs controlling a sound synthesis algorithm. This is a straightforward way of learning directly from data. As can be observed in the video, desired inputs can be specified along with their desired corresponding outputs and these data pairs can be encoded on a specific rule. Instead of specifying these data points in real-time moving faders, it would be straightforward to add a functionality to the FLCTK to learn them directly from a file on disk.

## 6. Incerta: An Acousmatic Multi-Channel Fuzzy Composition

*Incerta* is an 8-min acousmatic multi-channel composition created in MaxMSP with the FLCTK. Incerta is a latin word that could be translated into English as *vague*, in direct relation to the ability of fuzzy logic to handle uncertain data using vague concepts. The gist of the composition is very simple: twenty-one separate tracks of audio are presented in both temporal and spatial order according to a fuzzy logic inference engine.

The fuzzy system handles both the temporal and spatial presentation of the material across time. The twenty-one audio tracks are separated into three different groups, according to their pitch content, ranging from low-frequency textures to high pitches ones. Each group is presented at a given time on a specific spatial location.

**Figure 9:**
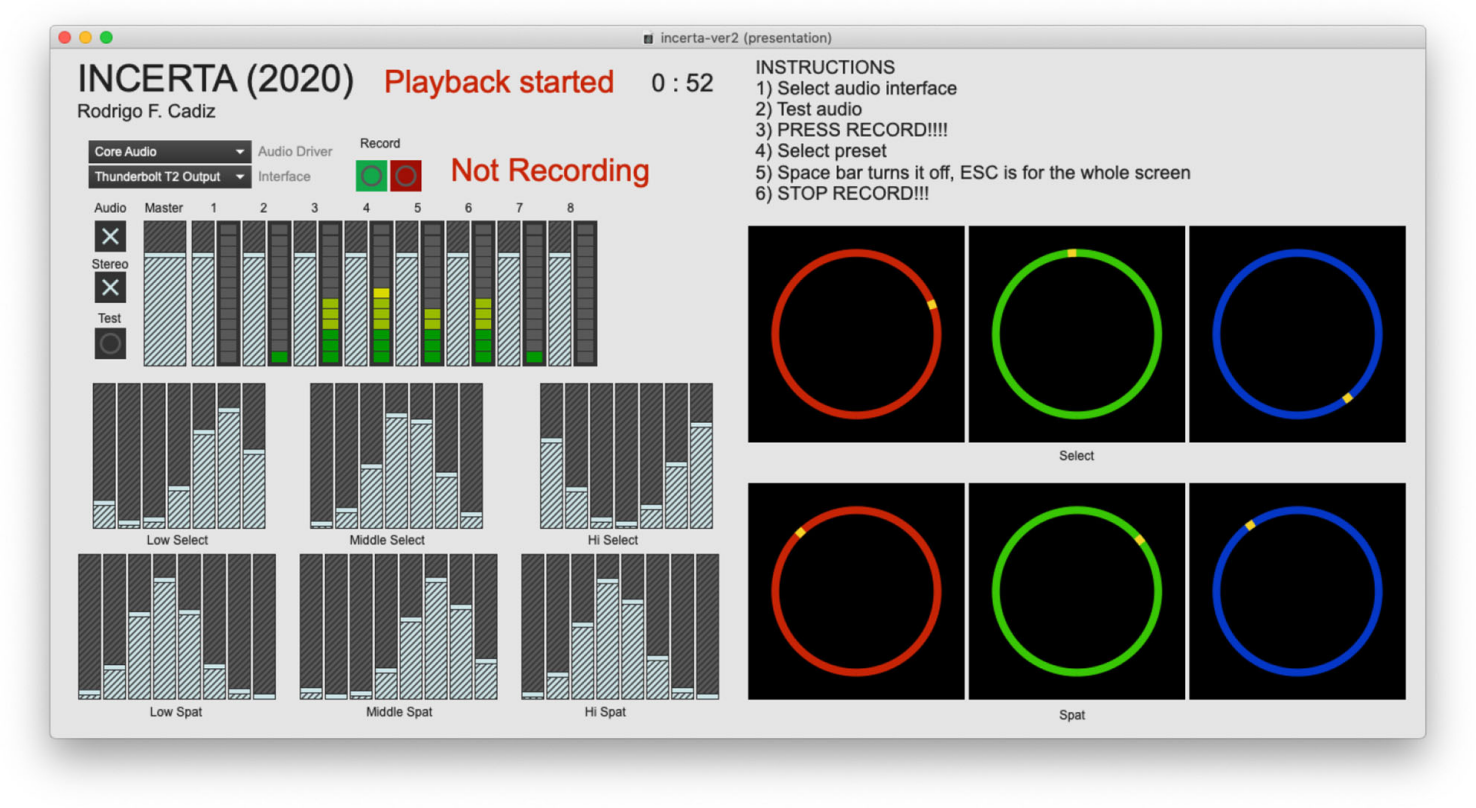
Screenshot of the main interface of *Incerta* at time 0:52. The six Gaussian curves for the control of the sound material selection and spatialization can be seen at the bottom left. Circles on the right displays the rotation angle that is used to specify the mean value of each Gaussian curve.

**Figure 10:**
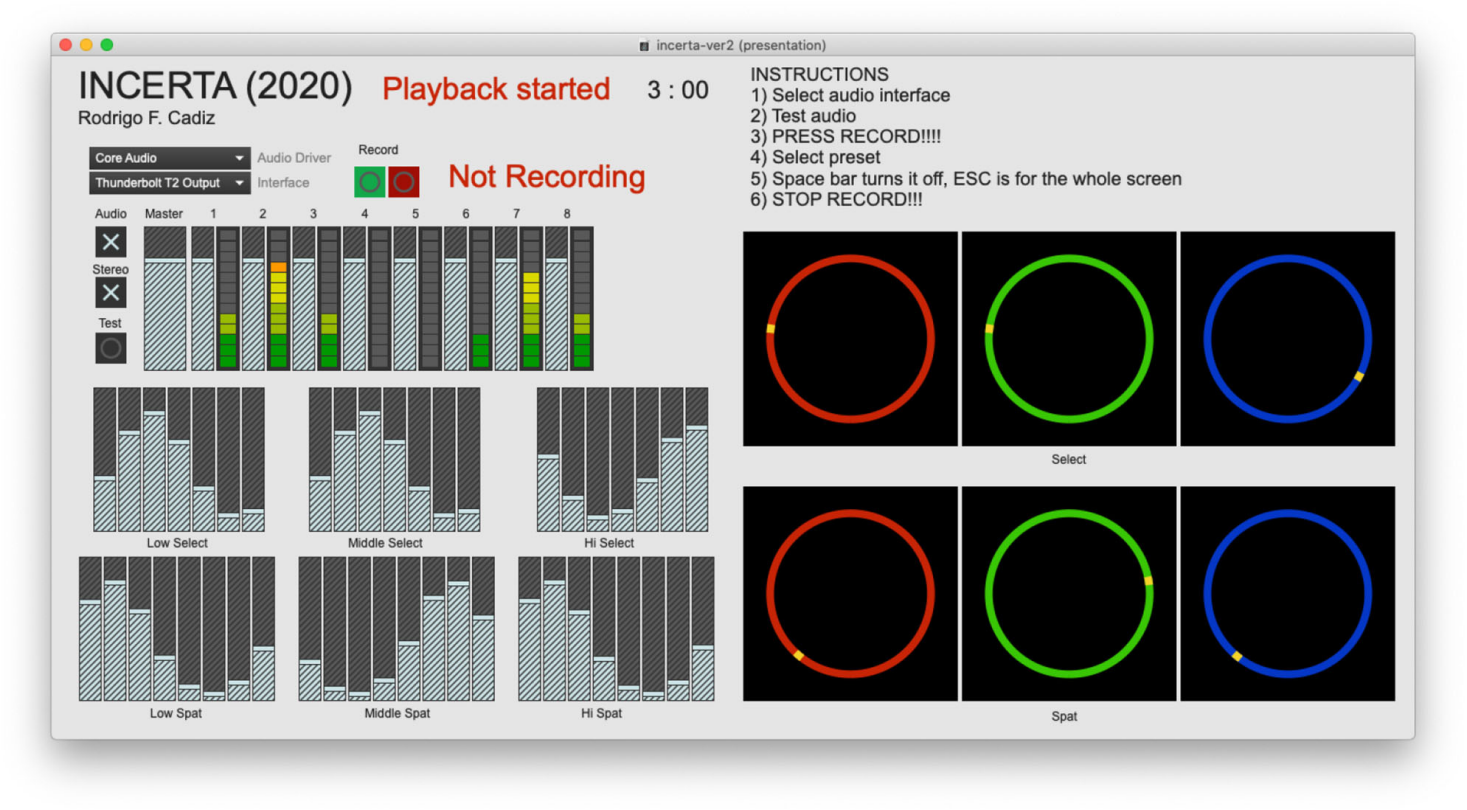
Screenshot of the main interface of *Incerta* at time 3:00. The six Gaussian curves for the control of the sound material selection and spatialization can be seen at the bottom left. Circles on the right displays the rotation angle that is used to specify the mean value of each Gaussian curve.

Both the selection of individual sound files and spatial position in an eight-speaker system are determined by the selection of a specific Gaussian curve that specifies the amplitudes of a group of faders, as shown in Figures 9, 10. There are three curves for each pitch content (low, medium, and high) and three additional curves for the circular spatial position of each group. The mean of each curve is controlled by an angle variable in such a way that the faders overlap circularly. The Gaussian curves can also be made wider or thinner, and thus affecting a different number of faders, by controlling their standard deviation.

The fuzzy system takes the rotation angle of each of the six Gaussian curves as inputs and also a time variable that allows for time-based behavior as time progresses. In total, there are seven inputs to the system. The outputs of the system are the change that each angle should experience at the next time step and two variables that control the standard deviation of the selection and spatial curves. This is an example of a closed feedback system, where some of the outputs of the system affect the inputs at the next time step.

The fuzzy variables used in this composition are described in Table 7 and can take the following values: Very Short (VSh), Short (Sh), Medium Short (MSh), Medium (M), Medium Large (MLa), Large (La), and Very Large (VLa) for time, Fast counter-clockwise (FCC), Medium counter-clockwise (MCC), Slow (Sl), Medium clockwise (MC), Fast clockwise (FC) for rotation angles and Very Low (VL), Low (L), High (H), and Very High (VH) for standard deviations.

**Table 7:** Input and output variables for *Incerta*, an acousmatic composition for eight channels.

**Input variables**	**Output variables**
Low pitch selection angle (2)	Change in low pitch selection angle (1)
Middle pitch selection angle (3)	Change in middle pitch selection angle (2)
High pitch selection angle (4)	Change in high pitch selection angle (3)
Low spatial selection angle (5)	Change in low pitch spatial angle (4)
Middle spatial selection angle (6)	Change in middle pitch spatial angle (5)
High spatial selection angle (7)	Change in high pitch spatial angle (6)
Time (1)	Selection curve standard deviation (7)
	Spatial curve standard deviation (8)

*Each of the variable numbers have been assigned a number in parenthesis, as shown in Table 8. There are seven inputs and eight outputs in totals*.

The rules for each system were created based on musical criteria, as shown in Table 8. In this approach to composition, most of the composer's work deals with the design and tuning of the fuzzy inference rules. Once the rules are established, the piece unfolds in real-time as the composer specified. Rules were designed in order. First, time dependence is established. Then, one rule for each possible fuzzy value of each one of the inputs is provided. This design methodology produces thirty-seven rules in total. Of course, these rules can be tweaked and fine-tuned to obtain specific desired behavior, but changing these rules too much would result perhaps in a different composition.

**Table 8:** Fuzzy rules for *Incerta*, an acousmatic composition for eight channels.

	**Inputs**							**Outputs**							
**Rule**	**1**	**2**	**3**	**4**	**5**	**6**	**7**	**1**	**2**	**3**	**4**	**5**	**6**	**7**	**8**
1	VSh	–	–	–	–	–	–	Sl	Sl	Sl	Sl	Sl	Sl	VL	VL
2	Sh	–	–	–	–	–	–	FC	FCC	FCC	FC	FCC	FC	L	L
3	MSh	–	–	–	–	–	–	FCC	Sl	MC	Sl	MC	Sl	M	M
4	M	–	–	–	–	–	–	Sl	Sl	Sl	Sl	Sl	Sl	H	H
5	MLa	–	–	–	–	–	–	FC	FCC	FC	FCC	FC	FCC	VH	VH
6	La	–	–	–	–	–	–	FC	FCC	FC	FCC	FC	FCC	M	M
7	VLa	–	–	–	–	–	–	Sl	Sl	Sl	Sl	Sl	Sl	VL	VL
8	–	FCC	–	–	–	–	–	FC	–	–	–	FC	–	–	–
9	–	MCC	–	–	–	–	–	Sl	–	–	–	–	–	–	–
10	–	Sl	–	–	–	–	–	MC	–	–	–	–	–	–	–
11	–	MC	–	–	–	–	–	FC	–	–	–	–	–	–	–
12	–	FC	–	–	–	–	–	FCC	–	–	–	FC	–	–	–
13	–	–	FCC	–	–	–	–	–	FC	–	–	FCC	–	–	–
14	–	–	MCC	–	–	–	–	–	FC	–	–	–	–	–	–
15	–	–	Sl	–	–	–	–	–	–	–	–	–	–	–	–
16	–	–	MC	–	–	–	–	–	FCC	–	–	–	–	–	–
17	–	–	FC	–	–	–	–	–	FCC	–	–	FCC	–	–	–
18	–	–	–	FCC	–	–	–	–	–	MC	–	FC	–	–	–
19	–	–	–	MCC	–	–	–	–	–	FC	–	–	–	–	–
20	–	–	–	Sl	–	–	–	–	–	FCC	–	–	–	–	–
21	–	–	–	MC	–	–	–	–	–	MCC	–	–	–	–	–
22	–	–	–	FC	–	–	–	–	–	Sl	–	–	–	–	–
23	–	–	–	–	FCC	–	–	–	FCC	–	MC	MCC	–	–	–
24	–	–	–	–	MCC	–	–	–	–	–	MC	–	–	–	–
25	–	–	–	–	Sl	–	–	–	–	–	FCC	–	–	VH	VL
26	–	–	–	–	MC	–	–	–	–	–	MCC	–	–	–	–
27	–	–	–	–	FC	–	–	–	–	–	MCC	MC	–	–	–
28	–	–	–	–	–	FCC	–	–	–	–	–	FC	–	–	–
29	–	–	–	–	–	MCC	–	–	–	–	–	FC	–	–	–
30	–	–	–	–	–	Sl	–	–	–	–	–	FCC	–	VL	VL
31	–	–	–	–	–	MC	–	–	–	–	–	FCC	–	–	–
32	–	–	–	–	–	FC	–	FCC	–	–	–	FCC	–	–	–
33	–	–	–	–	–	–	FCC	–	–	–	–	–	FC	–	–
34	–	–	–	–	–	–	MCC	–	–	–	–	–	MC	–	–
35	–	–	–	–	–	–	Sl	–	–	–	–	–	FC	VH	VH
36	–	–	–	–	–	–	MC	–	–	–	–	–	FCC	–	–
37	–	–	–	–	–	–	FC	–	–	–	–	–	MCC	–	–

*All rules have the same weight and are connected by AND operators. A “–” indicates that the value of the fuzzy variable can be anything. Variable names are specified in Table 7. The fuzzy values that the variables can take are: Very Short (VSh), Short (Sh), Medium Short (MSh), Medium (M), Medium Large (MLa), Large (La), Very Large (VLa), Fast counter-clockwise (FCC), Medium counter-clockwise (MCC), Slow (Sl), Medium clockwise (MC), Fast clockwise (FC), Very Low (VL), Low (L), High (H), and Very High (VH)*.

As time progresses the state of the whole fuzzy system changes, as it can be seen by comparing Figure 9 with Figure 10, which corresponds to the same instance of the piece at different times, 0:51 and 3:00, respectively. The position of each of the rotating circles is different, resulting in a different sonic output at those specific times. Another very interesting aspect of this approach is that the initial point of each input variable determines a different outcome. Even though there is some time dependence, the fact that there are closed loops in the system results in fuzzy outputs that are highly dependent on the initial conditions. As this composition is based on pre-generated sonic material, this complex behavior of the fuzzy system does not result in a totally different piece for different starting points, but there are indeed noticeable differences from one version to another. In this sense, this composition does not have a unique final format, but as many formats as there are initial conditions, which is infinite in theory.

The fuzzy system used in this piece can produce complex dynamic behavior, as it can be observed in the accompanying videos of three different performances or instances of the piece. The time evolution of each variable is distinct and the overall behavior of the piece is not the same. This is due to the thirty-seven inference rules encoded on the system. Videos of each of the *Incerta* performances can be found in the Supplementary Material.

## 7. Discussion and Conclusions

The provided examples show that the power of fuzzy systems lies in the parallel computation of very simple rules. A mathematical model is not needed to approximate any system, no matter how complex it could be. Fuzzy systems are, in general, much simpler to construct and use than other AI techniques, such as deep neural networks. They do not require a large amount of training or extremely large data sets. Rather, a few if-then like fuzzy inference rules, inspired by expert knowledge or common sense, are usually enough to develop interesting systems for musical creation. Fuzzy systems are very suitable tools for the control of high dimensional parameter spaces, as it could be observed from the algorithmic composition and parametric control examples, where five parameters could be successfully addressed with only two control dimensions. Also, because fuzzy systems can approximate any non-linear process, it is easy to create complex behavior, something highly valuable in creative endeavors.

Fuzzy logic is also a powerful way to implement non-linear mappings and intuitive control of non-intuitive synthesis parameters. However, one of the weaknesses of a fuzzy logic approach to parametric composition would be the time required to appropriately design adequate rules for the inference system. In engineering control applications, these rules are derived from expert knowledge or machine learning processes, where the rules are derived from trained data. In artistic applications, these rules constitute the heart of the underlying parameter mapping and it becomes really hard to select appropriate rules for a specific desired output when the parameter space is highly dimensional, which is often the case. Rule specification becomes an art form in itself, and it requires time and the development of expert knowledge specific to this kind of composition. In creative applications, when designing the fuzzy variables and rules, it is not necessary to worry about stability or controllable issues, the items on which control engineers spend most of their time. On the contrary, instability could be something very appealing to a composer.

The FLCTK constitutes a powerful and simple approach to the compositional control of computer music, as demonstrated by the examples described in this article. It has been successfully implemented in a variety of situations: algorithmic composition, particle-based synthesis, and granular synthesis control, and in the composition of a whole piece entitled *Incerta*. Overall, the FLCTK is a simple way of designing and implementing fuzzy logic inference systems inside MaxMSP. Its compatibility with MATLAB's fuzzy logic toolbox also allows this environment to be used in the design and test stages of the fuzzy models.

Finally, we would like to encourage the use of fuzzy systems as an alternative to the current trend of using deep learning and generative models for musical creation. Both approaches can complement each other. However, one big difference between these approaches is knowledge representation. In neural networks, it is sometimes very hard to understand what the knowledge captured by the network is. In fuzzy logic, it is very clear what is being learned and represented as all knowledge is encoded in the rules of the system, even if the rules were learned directly from data. This is a major difference between these approaches, and for some types of music, a fuzzy approach could be better suited than a purely data-based one.

## Data Availability Statement

All computer code and software tools generated for this study are included in the article/Supplementary Material.

## Author Contributions

The author confirms being the sole contributor of this work and has approved it for publication.

## Conflict of Interest

The author declares that the research was conducted in the absence of any commercial or financial relationships that could be construed as a potential conflict of interest.

## Footnotes

^1^http://cycling74.com

^2^https://www.mathworks.com/help/fuzzy/index.html

## Supplementary Material

Video S1 Algorithmic composition example.

Video S2 Sound synthesis example.

Video S3 Parametric control example.

Video S4 Many-to-many parameter control example.

Video S5 Incerta performance 1.

Video S6 Incerta performance 2.

Video S7 Incerta performance 3.
